# Prequestions do not enhance the benefits of retrieval in a STEM classroom

**DOI:** 10.1186/s41235-017-0078-z

**Published:** 2017-10-25

**Authors:** Jason Geller, Shana K. Carpenter, Monica H. Lamm, Shuhebur Rahman, Patrick I. Armstrong, Clark R. Coffman

**Affiliations:** 1 Department of Psychology, The University of Alabama-Birmingham, 415 Campbell Hall, 1530 3rd Avenue South, Birmingham, AL 35294-1170 USA; 2 Department of Psychology, Iowa State University, W112 Lagomarcino Hall, 901 Stange Road, Ames, IA 50011-1041 USA; 3 Department of Chemical and Biological Engineering, Iowa State University, 2114 Sweeney Hall, 618 Bissell Road, Ames, IA 50011-2230 USA; 4 Department of Genetics, Development, and Cell Biology, Iowa State University, 3258 Molecular Biology Building, 2437 Pammel Drive, Ames, IA 50011-1079 USA

**Keywords:** Prequestions, Retrieval practice, Classroom, Engineering education

## Abstract

Answering questions before a learning episode—“prequestions”—can enhance memory for that information. A number of studies have explored this effect in the laboratory; however, few studies have examined prequestions in a classroom setting. In the current study, the effects of prequestions were examined in an undergraduate course in chemical engineering. At the start of several class meetings, students were provided with a prequestion to answer about the upcoming lesson, and then were asked to provide ratings of confidence in their answers, familiarity with the content in the prequestion, and how much of the assigned reading they had completed. At the end of class, students were given the same question again (postquestion), along with a different question from the same lesson (new question). On a quiz at the end of each week, students were given the postquestions and new questions again, in addition to never-before-seen questions (quiz-only questions) from the same lessons. Performance on questions at the end of class revealed no difference in performance for postquestions vs. new questions. Although weekly quiz performance revealed an effect of retrieval practice—superior memory for material tested at the end of class (postquestions and new questions) compared to material not tested (quiz-only questions)—there was no difference in weekly quiz performance on postquestions vs. new questions. These results suggest that retrieval practice is beneficial to learning in the classroom. However, prequestions do not appear to enhance learning, nor to enhance the effects of retrieval practice.

**Electronic supplementary material:**

The online version of this article (doi:10.1186/s41235-017-0078-z) contains supplementary material, which is available to authorized users.

## Significance

An important question in education is how to improve students’ learning of course concepts. Asking students questions over what they are learning—i.e., retrieval practice—is an effective way to enhance retention of concepts. However, cognitive psychology research has not always provided clear and concrete recommendations for how instructors can incorporate this technique into their teaching. The current study shows that “clicker” questions administered at the beginning and end of class can provide a straightforward and effective way to enhance students’ learning in chemical engineering. In particular, material that was tested at the end of class was remembered 30% better on a later review quiz, relative to material from the same lesson that was not tested. Asking questions at the beginning of class—i.e., “prequestions”—and repeating them at the end of class, however, did not further this benefit. Thus, when instructors are faced with the question of when to incorporate clicker questions into a given day’s class—at the beginning, at the end, or both—the current results suggest that such questions would be best placed at the end of class. These results provide a simple yet effective way that instructors can incorporate retrieval practice into their teaching.

## Background

A common belief amongst students and teachers is that testing serves primarily as an evaluative tool, as a “dipstick” to assess what one knows and does not know (Hartwig & Dunlosky, 2012; McAndrew, Morrow, Atiyeh, & Pierre, 2016; Morehead, Rhodes, & DeLozier, 2016). However, there is a voluminous literature demonstrating, both in the laboratory and in the classroom, that taking an initial test over previously encountered material actually potentiates learning compared to simply restudying the material. This common and robust finding has been referred to as the *testing effect*, or *retrieval practice* (for recent reviews, see Carpenter, 2012; Dunlosky, Rawson, Marsh, Nathan, & Willingham, 2013; Karpicke, in press; Kornell & Vaughn, 2016; McDermott, Arnold, & Nelson, 2014; Roediger, Putnam, & Smith, 2011; Rowland, 2014).

Classroom instruction could benefit greatly from this easy to implement and inexpensive technique (Roediger & Pyc, 2012). Science, technology, engineering, and mathematics (STEM) classrooms, in particular, are in need of instructional approaches that can promote student achievement, as students struggle to gain proficiency and have increased anxiety over the subject matter (Ackerman, Kanfer, & Beier, 2013; Chen, 2013). Toward this goal, in recent studies retrieval practice has been shown to potentiate student learning in courses such as biology, biochemistry, chemistry, and engineering (Butler, Marsh, Slavinsky, & Baraniuk, 2014; Carpenter et al., 2016; Horn & Hernick, 2015; Pyburn, Pazicni, Benassi, & Tappin, 2014).

While the retrospective benefits of testing have been clearly demonstrated, an open question concerns the *prospective* benefits of testing. That is, does the positive effect of testing also extend to testing that takes place before exposure to material that must be learned? There is evidence that giving tests prior to learning improves memory for that information. In a typical study using these “prequestions”, individuals read passages of text and then complete a test over the material. Half the participants receive prequestions before each to-be-read segment (i.e., the prequestion group), and the other half read each segment without receiving pre-questions (i.e., the control group). On the final test, the prequestion group typically outperforms the control group (Little & Bjork, 2016; Peeck, 1970; Pressley, Tanenbaum, McDaniel, & Wood, 1990; Richland, Kornell, & Kao, 2009; Rickards, Anderson, & McCormick, 1976).

In these studies, corrective feedback was not provided to students at the time they answered the prequestions. Instead, to learn the information students had to discover it while they read the material. Even though participants often got the prequestion wrong—because they had not yet learned the information—the better learning that occurred in the prequestion group relative to the control group demonstrates that students can successfully discover information needed to answer the prequestion, and can retain this information at a later time. Students who receive prequestions typically show better memory for portions of the passage that were relevant to the prequestions—i.e., *prequestioned* information—compared to other portions of the passage that were not relevant—i.e., *non-prequestioned* information (e.g., Bull & Dizney, 1973; Pressley et al., 1990; Richland et al., 2009). In addition, some research shows that under some circumstances the prequestion group retains non-prequestioned information better than the control group (Carpenter & Toftness, 2017), suggesting that prequestions may serve to enhance overall processing of the material.

Given its simplicity and apparent effectiveness, the prequestion technique would seem to be a useful tool that can be implemented to enhance student learning in instructional settings. Indeed, a Department of Education-sponsored practice guide for educators lists prequestions as one of a handful of evidence-based techniques, judged by a panel of experts on learning, that are concrete and highly applicable to education (Pashler et al., 2007). Though the benefits of prequestions have been consistently demonstrated in laboratory studies with reading materials, the expert panel noted that the level of ecologically valid evidence supporting the prequestion technique has been low. In particular, they concluded that “there is little or no published experimental evidence regarding whether pre-questions will promote the learning of orally presented classroom content,” (p. 19).

Indeed, in the 10 years since the publication of the practice guide, the potential of prequestions to enhance learning in educational settings has remained largely unexplored. To date, only one known study has explored prequestions in a classroom setting. McDaniel, Agarwal, Huelser, McDermott, and Roediger (2011) (experiments 2a and 2b) presented middle school science students with questions at the start of each lesson and end of each lesson (prelesson-postlesson questions), or only at the end of each lesson (postlesson questions). Across two experiments, they found mixed evidence for a benefit of prequestions. On the postlesson questions, performance was generally higher for material that had been prequestioned (i.e., prelesson-postlesson questions) compared to material that had not been prequestioned (i.e., postlesson questions). There was a numeric, but non-significant, advantage in experiment 2a (78 vs. 76%, respectively, *d* = 0.15), and a modest but significant advantage in experiment 2b (84 vs. 79%, respectively, *d* = 0.35). Thus, contrary to the results of laboratory studies where the benefits of prequestioned information over non-prequestioned information have been quite large—e.g., 25% in the study by Richland et al. (2009) (*d* = 1.70)—these effects appear to be much smaller in classroom settings.

Such a reduction in the size of the effect between laboratory and classroom settings is not particularly surprising given a multitude of factors that can be controlled in the laboratory but not in the classroom (e.g., see Butler et al., 2014). Potential sources of unexplained variance in course settings could include students’ prior knowledge of the material, out-of-class studying, and individual differences in interest, motivation, and academic achievement.

Beyond these factors, differences in the way that prequestions are administered could lead to different effects on learning as well. In particular, the benefits of prequestions could be reduced under conditions in which the mechanism(s) believed to be responsible for these effects are less likely to be engaged. Theoretical discussions of the prequestion effect have included the (non-mutually exclusive) possibilities that prequestions stimulate curiosity (e.g., Berlyne, 1954, 1962), that they increase attention to those parts of the material that are relevant to the prequestions (Peeck, 1970; Pressley et al., 1990), or that they provide a metacognitive “reality check” (e.g., Bjork, Dunlosky, & Kornell, 2013) that gives students a clear realization that they do not know the answers and must allocate attention and effort to discover them. Such processes are most likely to occur under conditions in which students’ curiosity to know the answers to the prequestions is high, they are attentive to the material, and they can successfully discover the answers to the prequestions during the learning episode.

These things considered, it is quite possible that the effects of prequestions are consistently limited in classroom settings. Compared to a laboratory setting, a classroom is likely to involve presentation of information that is more lengthy and complex. During a 50-minute (or longer) class period, students may have difficulty sustaining attention and noticing information that is relevant to a prequestion that was asked at the beginning of class. Even if curiosity to know the answer is high, this rather lengthy duration of time may introduce interruptions in the processing of the prequestion and the ease with which students can remember it and connect it to the relevant information in the lesson.

In the current study, we set out to provide additional data on the effectiveness of prequestions in classroom settings. In a college-level course on chemical engineering, students were asked a question at the beginning of several class meetings that pertained to that day’s lesson. At the end of class, they were asked the same question again, in addition to a different question from the same lesson. Consistent with laboratory studies on prequestions, we did not provide students with feedback of the correct answers at the time of the prequestions. The answer to a given prequestion was always contained in the class lesson that immediately followed, and to learn the answer students needed to discover it during class. This was somewhat different from the study by McDaniel et al. (2011), in which feedback was provided after the prequestions and the lessons relevant to some of the prequestions occurred during subsequent class meetings (these were necessary design features of the study that aligned with the way in which the course was structured). However, common to the current study and that of McDaniel et al. (2011) was the fact that prequestions were administered in real classrooms where the duration of a lesson was longer than the duration of a typical laboratory experiment. If the length and complexity of information presented in authentic course environments is a contributing factor to the attenuation of prequestion effects, then the current study might be expected to yield results similar to those of McDaniel et al. (2011). To the extent that feedback is important to these effects—such that withholding feedback produces benefits of prequestions, perhaps due to enhanced curiosity (e.g., Berlyne, 1954, 1962)—then the current study might be more likely to yield benefits of prequestions.

Given the variability among students in classroom environments, we also explored the potential role of individual differences in the prequestion effect. We collected information about students’ academic achievement (i.e., grade point average (GPA)), in addition to their confidence in their answers to the prequestions, familiarity with the information in the prequestions, and how much of the assigned reading they had completed prior to class. Such individual differences have not been examined in any of the known research on prequestions, but could be of theoretical and practical importance. In particular, the idea that prequestions provide a metacognitive reality check that reduces students’ overconfidence could be tested by examining the relationship between students’ confidence in their answers to the prequestions and their later accuracy on those questions at the end of class. A negative relationship between confidence and later accuracy (other factors controlled) might offer some support for this notion, in that students who are less certain about their knowledge of the concept in the prequestion are more likely to learn and retain the answer to that question when it is encountered in class.

Abundant research on retrieval practice shows that testing students over material they have encountered boosts memory retention (e.g., Carpenter, 2012; Dunlosky et al., 2013; Karpicke, in press; Kornell & Vaughn, 2016; McDermott et al., 2014; Roediger, Agarwal, McDaniel, & McDermott, 2011; Rowland, 2014). However, it is unknown whether these benefits are enhanced by giving students a chance to answer the test questions (without feedback) before encountering the material during class. To examine this, we gave students a practice quiz at the end of each week of the course. The quiz included the questions that were asked at the beginning and end of class, along with questions that were asked only at the end of class, and never-before-seen questions that were drawn from the same lessons. This provided the opportunity to examine delayed retention for material that was tested vs. material that was not tested—i.e., retrieval practice—and to determine whether this effect was stronger for material that was prequestioned before it was tested at the end of class.

Thus, the current study provided a classroom investigation of the effects of prequestions on learning course information. In addition, we examined whether or not these effects are influenced by individual differences in students’ confidence, academic achievement, and out-of-class preparation. Finally, we examined whether the common benefits of retrieval practice—superior long-term retention for information that has been tested—could be enhanced by providing students with prequestions prior to the lesson.

## Methods

### Participants

Participants in this study were recruited from 77 students enrolled in a chemical engineering course at Iowa State University (ISU). The study protocol was reviewed and approved by ISU’s Institutional Review Board (IRB). At the beginning of the semester, a researcher visited class and informed students about the study. All students were invited to participate by providing informed consent that allowed their scores on the course-related activities to be analyzed and information about their grade point average (GPA) to be collected for research purposes. Throughout the semester, three students dropped the course and 12 students declined to have their data included in the study, leaving 62 students in the sample.

### Course setting

The course was a Material and Energy Balances course for second-year chemical engineering majors. The course covered three content modules: (1) material balances, (2) volumetric properties and multiphase systems, and (3) energy balances. Following the content modules, there were two modules devoted to case studies. The course met three times per week for 50 minutes over a 15-week semester. There were ten class meetings dedicated to instructional activities for each of the three content modules. Other class meetings were dedicated to scheduled exams, review of information following exams, and case studies.

The course was structured using the team-based learning instruction method (Michaelsen, Knight, & Fink, 2004; Sibley, Ostafichuk, Roberson, Franchini, & Kubitz, 2014). One to two hours of out-of-class work per class meeting were required, which included assigned reading and homework problems. During each class period the learning activities included some mini-lectures by the instructor to clarify concepts from the reading, and application exercises that were solved by student learning teams. The application exercises were designed to provide practice at applying the concepts that students were learning, and these increased in complexity and difficulty as the content module progressed. There were three individual examinations, each administered at the end of a content module.

### Materials and design

Within each of the three content modules in the course, there were five lesson days on which students were asked prequestions at the beginning of class. These 15 class meetings were intentionally selected to coincide with the days in each content module where the lessons were aimed at helping the students learn concepts that they would later use for more challenging problem-solving and application exercises. These 15 lessons consisted of a mixture of instructor presentation, some in-class problem-solving exercises, and discussion by the instructor to clarify the concepts being learned. The other class periods throughout the semester (on which prequestions were not asked) consisted primarily of hands-on problem-solving exercises or scheduled activities related to exams. Thus, the prequestions took place on days that were well-suited for the acquisition of new information that students needed for later stages of the course.

For each of the 15 target class meetings, a set of three questions was selected to align with the content for that day. These questions assessed knowledge of concepts that were included in the assigned readings for each class meeting, although the exact questions themselves were not included in the readings. Each set of three questions contained a prequestion, a new question, and a quiz-only question. The prequestion was asked at the beginning of class and repeated at the end of class, whereas the new question was asked only at the end of class. This allowed us to measure retention at the end of class for prequestioned vs. non-prequestioned information. To explore whether prequestions enhance the effects of retrieval practice, an online quiz was given to students at the end of each week. This online quiz contained both the prequestion and the new question from that week, along with a third question from the same lesson that had not been asked before—i.e., the quiz-only question. This allowed us to explore the delayed effects of questions that had been asked at the beginning and end of class (prequestions), versus questions asked only once at the end of class (new questions), and to compare performance on these questions to performance on questions from the same lesson that had not been asked before (quiz-only questions). Better performance on new questions vs. quiz-only questions would demonstrate the benefits of retrieval practice. Better performance on prequestions vs. new questions would demonstrate that the effects of retrieval practice are bolstered by providing an opportunity to answer the questions at the beginning of class.

In this real classroom environment, questions could not be randomly assigned or counterbalanced across question type. Therefore, we ensured that the relevant properties of the questions were controlled to the extent possible. All of the questions were selected from the AIChE Concept Warehouse (Koretsky et al., 2014)—a database containing concept questions for several core subjects in the chemical engineering discipline and information about each question’s difficulty based on student performance in the course(s) in which it had been previously used. In the current study, the instructor chose three questions from the database that aligned with the material for each of the 15 target class meetings. All three questions for a given lesson were multiple-choice, relevant to that day’s lesson, assessed independent concepts (such that knowing the answer to one question would not facilitate knowing the answer to another question), and were reasonably matched for difficulty. The difficulty index for a given question—i.e., the proportion of previous respondents who answered the question correctly—could be matched exactly for the three-question set pertaining to some lessons. For other lessons, the pool of available questions did not contain a sufficient number that allowed an exact match in difficulty across the three-question set. In the cases where question difficulty could not be matched exactly, based on the assumption that students would perform best on questions they had seen most often and worst on questions they had seen least often, we arranged the questions so that the hardest question appeared as the prequestion and the easiest question appeared as the quiz-only question. The difference in difficulty across a given three-question set was never higher than 0.10 from the prequestion to the new question, and never higher than 0.30 from the prequestion to the quiz-only question. To control for any potential effects of question difficulty, we entered question difficulty as a covariate in the analyses comparing performance across question types.

**Fig. 1:**
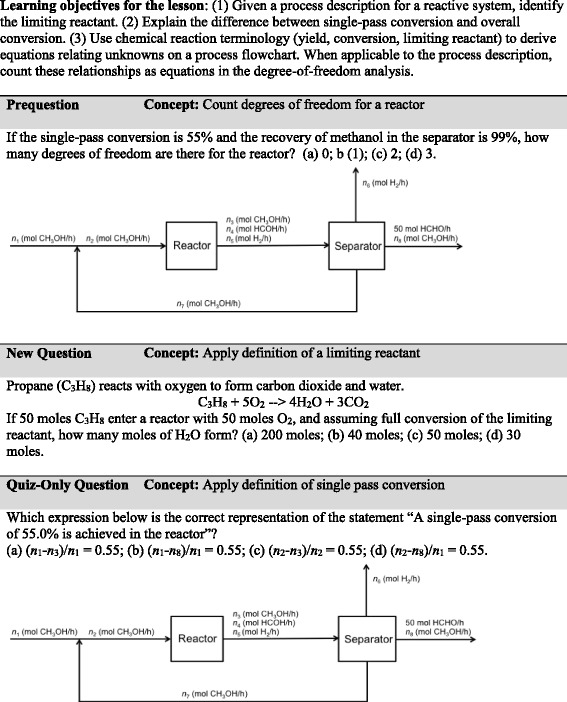
Representative three-question set for a lesson (adapted from Koretsky et al., 2014). This example illustrates the alignment of questions with the learning objectives for the lesson and the independence between questions

Figure 1 shows a representative three-question set. This lesson pertains to the topic of material balances for processes with a chemical reaction. The concepts covered included counting the degrees of freedom in a reactor, applying the definition of a limiting reactant, and applying the definition of single pass conversion.

### Procedure

During the 15 target class meetings, the instructor presented a slide at the beginning of class to announce that class would begin with a short series of questions to gauge students’ understanding of the lesson topic for that day. Students were reminded that the answers to the questions would not be graded and that they would earn one course point for every response submitted. Students were encouraged to answer each question honestly and independently because the information collected would be used for future lesson planning. This brief introduction was repeated during each of the 15 target class meetings.

Immediately following the introduction, the instructor presented the slide containing the prequestion. Students indicated their responses to the prequestion using their individual response systems (“clickers”). The time limit for responding was set so that once the response rate reached 90%, the remaining students had 5 s to input an answer. Most prequestion responses were completed in under one minute. No feedback was provided about the correct answer to the prequestion or the distribution of responses. Immediately after the prequestion, students were presented with individual slides assessing their confidence in their answers, familiarity with the concept in the prequestion, and completion of the reading assignment. The confidence question inquired “How confident are you in your answer?” with response options A) very confident, B) confident, C) somewhat confident, or D) not at all confident. The familiarity question inquired “Which statement best describes your familiarity with the question you just answered?” with response options A) very familiar: I knew this concept before class, B) somewhat familiar: I have seen this concept, but I do not remember the details, C) Somewhat unfamiliar: I think I have seen this concept, but I do not remember it, or D) very unfamiliar: this concept is new to me. The reading assignment question inquired “How much of the reading assignment did you complete prior to today’s class?” with response options A) all of it, B) more than half, C) less than half, or D) none of it. Students answered each of these questions one at a time with individual clickers.

**Fig. 2:**
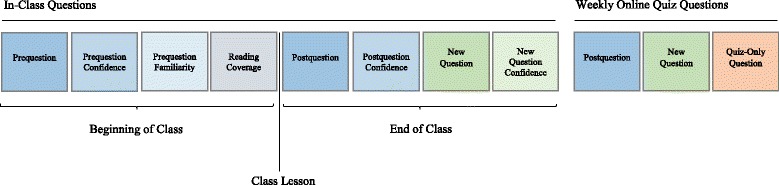
In-class questions and weekly quiz questions. At the beginning of each class meeting, students answered one prequestion over the upcoming lesson, followed by their confidence in their answer, familiarity with the material in the question, and how much of the reading assignment they completed prior to class. At the end of the class meeting, students answered the same question as before and rated their confidence again, and also answered a new, never-before-seen question from the same lesson and rated their confidence. After students’ confidence ratings on the end-of-class questions, the instructor provided the answers. On the weekly online quiz, students answered the same postquestion and new question, along with a never-before-seen (i.e., quiz-only) question from the same lesson, and received correct answer feedback

A schematic of the procedure is shown in Fig. 2. Immediately following the prequestion, confidence, familiarity, and reading ratings, the instructor commenced with the class lesson for that day. This began with a presentation of the learning objectives and an invitation for students to raise any questions about the assigned reading for that day’s lesson. If students asked questions, the instructor provided a 3 to 5 minute mini-lecture to clarify concepts from the reading. Students never requested the answer to the prequestion, nor did they ask a question that specifically related to the prequestion. After the mini-lecture, the instructor provided one or more team-based learning activities (typically worksheets with questions to answer or short problems to solve) aimed at reinforcing the learning objectives for that lesson.

Near the end of the team-based learning session, teams reported their answers to the class and the instructor provided closing remarks to summarize and conclude the team activities. During the last segment of the class period (i.e., approximately 10 minutes before the end of class), the instructor presented the prequestion again, with the answer choices arranged in a new, unique order that was different from what students saw at the beginning of class. After students answered the question with their clickers, the confidence question was presented in the same format as at the beginning of class. After all confidence responses were collected, the instructor presented the correct answer to the prequestion and provided a brief explanation. Next, the new question was presented, followed by the confidence question. After all responses were collected, the instructor presented the correct answer to the new question and provided a brief explanation.

The slides for the prequestion and new question sequence were kept in a separate file from the slides used for the lesson. Although students were provided access to the lesson slides outside of class, they were not directly provided with the questions that were used as prequestions and new questions. Aside from the class period in which these questions were administered, students were provided with access to these questions again only on the weekly quizzes.

Each week except for those that contained exams, students were required to complete a weekly quiz on the online course management system (Blackboard Learn). This quiz contained the prequestion, the new question, and the quiz-only question, in that order, for any of the class periods during that week that implemented the prequestion routine. The answer choices for the prequestion and the new question were arranged in an order that was different from any previous order in which they appeared (when they were presented in class). Additional questions designed to be more challenging were included in the weekly quiz, at the instructor’s discretion, and these other questions (usually story problems that required application of more than one concept) appeared after the prequestion, new question, and quiz-only questions. The quiz was released to students immediately after the last class meeting on Friday of each week, and was due by Sunday at 10 pm.

The online quizzes were not graded for correctness. Students received one point per answer for the first attempt for every quiz question that they answered by the deadline. Once a student submitted the quiz, they received a performance report that showed each question, their response to each question, the correct response to the question, and feedback from the instructor to explain the reasoning behind the correct answer. Once a weekly online quiz was released, it remained available to students for the duration of the semester and students could return to the quizzes as often as they wished to review the content. For data analysis purposes, we assessed quiz performance on only the first attempt at answering the prequestions, new questions, and quiz-only questions.

The first of the 15 target class meetings (during week 1) was used as a practice opportunity. During this meeting the instructor familiarized students with the in-class question procedure, ensured that clickers were working properly, and reminded students to complete the first weekly quiz before the deadline. Data from this practice session were not analyzed, leaving 14 class meetings (beginning in week 2) that were entered into the analyses.

## Results

### Scoring and pre-analyses

Students’ responses to the prequestions, new questions, and quiz-only questions were scored as either correct or incorrect. Students’ responses to the beginning-of-class questions assessing confidence, familiarity, and reading were re-coded (from A, B, C, or D) to numeric ordinal responses (1, 2, 3, or 4) such that higher numbers corresponded to greater degrees of confidence, familiarity, and reading. For each student we calculated the proportion of questions answered correctly on both the in-class questions and the online weekly quiz questions across the 14 target lessons. We also calculated students’ mean confidence, familiarity, and reading ratings across these lessons.

### Data analysis approach

Alongside traditional null hypothesis significance testing (NHST), we also report the Bayes factors for all nonsignificant comparisons. Unlike NHST that does not allow one to measure support that there is a true null effect, Bayes factors allow one to provide evidence for one model relative to another model. In this case, model 0 is the null hypothesis that there is no difference between conditions, and model 1 is the alternative hypothesis that there is a difference. The notation BF_01_ is used to express the probability of the data given the null hypothesis (model 0) relative to the alternative hypothesis (model 1). Jefferys (1961) provides a general heuristic for interpreting Bayes factors, in that a Bayes factor less than 3 is taken as weak evidence in favor of the null, and a Bayes factor greater than 3 is taken as substantial evidence in favor of the null.

### The effect of prequestions on retention of information from class

For in-class questions, data were included for a given lesson only if students completed all three questions for that lesson—the prequestion at the beginning of class, the same question repeated at the end of class (referred to here as the postquestion), and the new question appearing only at the end of class. Across students, the average number of lessons (out of the total 14) that received responses on all three questions was 11.37 (standard deviation (SD) = 2.48). Three students did not provide enough responses to contribute data for the three-question set associated with at least one lesson, resulting in 59 students entered into the following analyses.

For each student, we calculated the proportion of questions answered correctly out of the lessons for which that student responded to all three questions. Given differences in the overall mean difficulty (from the AIChE Concept Warehouse) across prequestions (M = 0.56, SD = 0.02) and new questions (M = 0.62, SD = 0.02) associated with these lessons, we entered question difficulty as a covariate in the analyses comparing question types.

**Fig. 3:**
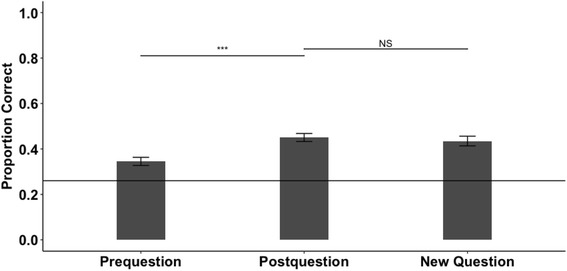
Proportion correct on in-class questions as a function of question type. Prequestion denotes questions asked at the beginning of class, postquestion denotes the same questions repeated at the end of class, and new question denotes new, never-before-seen questions from the same lessons asked at the end of class. *Horizontal line* denotes chance performance on the prequestions. *Error bars* represent standard errors. ***p < 0.001; *NS* not significant

Figure 3 shows the unadjusted mean proportion correct on the prequestions, postquestions, and new questions. Based on the prequestions completed (most of the 14 questions contained four alternatives, but three questions contained only three alternatives), chance performance was calculated at 26.5% (SD = 0.60%). Students’ performance on the prequestions (M = 0.35, SD = 0.15) was significantly greater than chance (*t*(58) = 4.01, *p* < 0.001, *d* = 0.74), reflecting some prior knowledge of the material at the time of the prequestions.

Comparison of performance on prequestions vs. postquestions indicated that students improved significantly at answering the same question from the beginning of class to the end of class (*t*(58) = 4.50, *p* < 0.001, 95% confidence interval (CI) [0.058, 0.15], *d* = 0.65). However, there was no significant difference in performance at the end of class on the postquestions vs. the new questions (*t*(58) = 1.52, *p* = 0.13, 95% CI [−0.029, 0.219], *d* = .35, BF_01_ = 3.20^1^), indicating that students did not show greater memory for prequestioned information relative to non-prequestioned information. Question difficulty did not significantly influence performance in either of these comparisons (*t*s < 1.43, *p*s > 0.15, BFs_01_ > 3).

### Predictors of prequestion effectiveness

We assessed the degree to which the effectiveness of prequestions could be influenced by individual differences in students’ confidence, familiarity, out-of-class reading, and GPA. This analysis required students to provide responses on all of the in-class questions, including the prequestion, postquestion, new question, corresponding confidence ratings, and ratings for familiarity and out-of-class reading. Fifty-nine students contributed responses on all questions for at least one lesson, and across students the average number of lessons (out of the total 14) receiving responses to all of these questions was 10.51 (SD = 2.65).

On average, students found the prequestions to be somewhat familiar, with an average rating of 3.11 (SD = 0.43). The average rating for out-of-class reading was 3.06 (SD = 0.66), indicating that students completed just over half of the assigned reading per lesson. Confidence ratings increased significantly from prequestion (M = 2.70, SD = 0.43) to postquestion (M = 2.97, SD = 0.39): *t*(58) = 7.04, *p* < 0.001, *d* = 0.66. At the end of class, confidence ratings were also higher for postquestions compared to new questions (M = 2.60, SD = 0.45; *t*(58) = 8.92, *p* < 0.001, *d* = 0.88).

A multiple regression analysis was conducted with postquestion accuracy as the dependent variable, and five predictors: accuracy on the prequestion, confidence on the prequestion, GPA, familiarity with the prequestion, and out-of-class reading. Table 1 shows the results. The five predictors accounted for a significant amount of variance in postquestion accuracy (*R* ^*2*^ = 0.20, *F*(5, 51) = 2.64, *p* = 0.034). The only predictor to emerge as significant was prequestion accuracy (*β* = 0.45, standard error (SE) = 0.14, *p* = 0.001), indicating that students were more likely to answer the postquestion correctly if they had answered the prequestion correctly. No evidence emerged that prequestion confidence, or any of the other predictors, was significantly related to postquestion accuracy. Bayesian analyses, however, revealed weak evidence in favor of these null effects (BFs_01_ < 3).

**Table 1:** Results of multiple regression analysis predicting postquestion accuracy

Predictors	*β*	SE	*p*	BF_01_	*R* ^*2*^
					0.20*
Prequestion accuracy	0.447*	0.137	0.001	0.032	
Prequestion confidence	0.102	0.063	0.528	1.96	
GPA	0.131	0.035	0.330	1.54	
Familiarity	−0.134	0.064	0.415	1.73	
Reading	0.068	0.035	0.626	2.08	

Analysis excludes two students for whom GPA was not available. *p < 0.05. The Bayes factor for each predictor reflects evidence for the null based on the full model compared to a model without the predictor (Rouder & Morey, 2012). The Bayes factor package in R (Rouder & Morey, 2012) was used to calculate Bayes factors in the multiple regression model

The same regression analysis was conducted with new question accuracy as the dependent variable. None of the predictors accounted for a significant amount of variance in new question accuracy (*t*s < 1.30, *p*s > 0.20), with Bayesian analyses again suggesting weak evidence in favor of the null effects (BFs_01_ < 3).

### The effect of prequestions on retrieval practice

The final question of interest was whether prequestions enhance the effects of retrieval practice. The weekly quizzes contained questions that students had seen twice before (once at the beginning of class and once at the end, referred to here as postquestions), had seen once before (new questions), or had never seen before (quiz-only questions). The benefits of retrieval practice would be reflected in an advantage of new questions over quiz-only questions. If prequestions add to this benefit, then weekly quiz performance would be greater for postquestions compared to new questions.

Analysis of performance on the weekly quiz questions required that students complete all three questions on the weekly quiz, in addition to the previous in-class questions corresponding to those same lessons. For example, if a student completed the three online quiz questions for a given lesson (the postquestion, new question, and quiz-only question associated with week 6), these data were only included if the student had also completed the three in-class questions for that same lesson (the prequestion, postquestion, and new question associated with week 6). Across students, the average number of lessons (out of the total 14) receiving responses on all six questions was 10.44 (SD = 2.82). Five students did not provide enough responses to contribute data for the six-question set associated with at least one lesson, resulting in 57 students entered into the following analyses.

For each student, we calculated the proportion of questions answered correctly out of the lessons for which that student responded to all six questions. Given differences in the mean difficulty (from the AIChE Concept Warehouse) across postquestions (M = 0.56, SD = 0.03), new questions (M = 0.61, SD = 0.03), and quiz-only questions (M = 0.68, SD = 0.04) associated with these lessons, we entered question difficulty as a covariate in all of the following comparisons.

**Fig. 4:**
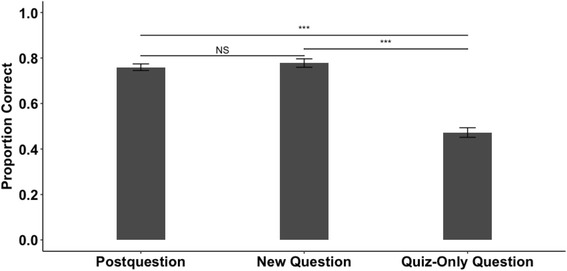
Proportion correct on weekly quizzes as a function of question type. *Postquestion* denotes questions asked at the beginning and end of class, *New Question* denotes questions asked only at the end of class, and *Quiz-Only Question* denotes never-before-seen questions from the same lessons that only appeared on the weekly quizzes. *Error bars* represent standard errors. ***p < 0.001; *NS* not significant

Figure 4 shows the unadjusted mean proportion correct on the weekly quizzes for postquestions, new questions, and quiz-only questions. Consistent with the benefits of retrieval practice, testing students at the end of class boosted performance on those same questions on the weekly quizzes. That is, students performed better on new questions compared to quiz-only questions (*t*(56) = 6.38, *p* < 0.001, 95% CI [0.190, 0.362], *d* = 1.35) and better on postquestions compared to quiz-only questions (*t*(56) = 4.03, *p* < 0.001, 95% CI [.132, .389], *d* = 0.93). However, students did not perform better on postquestions compared to new questions (*t*(56) = 0.77, *p* = 0.44, 95% CI [−0.043, 0.097], *d* = 0.16, BF_01_ = 7.20), indicating that prequestions did not enhance the benefits of retrieval practice. Question difficulty did not significantly influence performance in any of these comparisons (*t*s < 1.60, *p*s > 0.11, BFs_01_ > 2.8).

## Discussion

The current study contributes new data on the effects of prequestions in classroom settings. Asking a question at the beginning of class did not enhance learning of that information, relative to other information from class that was not prequestioned. These results are consistent with those of McDaniel et al. (2011) showing that prequestions produced no benefits on learning in one study (experiment 2a) and only minimal benefits in another study (experiment 2b). Contrary to the results of several laboratory studies (Carpenter & Toftness, 2017; Peeck, 1970; Little & Bjork, 2016; Pressley et al., 1990; Richland et al., 2009; Rickards et al., 1976), therefore, it appears that prequestions do not produce consistent and reliable effects on learning in classroom environments.

Nor do they appear to enhance the effects of retrieval practice. On the weekly quizzes, the benefits of retrieval practice were apparent, with performance highest for material that was tested at the end of class compared to material that was not tested. Performance did not differ, however, for material that was tested at the beginning and end of class compared to material that was tested only at the end.

Thus, information that was tested twice was not remembered better on a later quiz, compared to information that was tested only once. This could be due in part to the fact that feedback was not provided at the time of the prequestion. When students are uncertain about the correct answer, the most potent learning experience is likely to come from corrective feedback. Assuming that the learning gains from practice questions would be enhanced when feedback is provided (Carpenter, Sachs, Martin, Schmidt, & Looft, 2012; Fazio, Huelser, Johnson, & Marsh, 2010; Finn & Metcalfe, 2010; Kang et al., 2011; Pashler, Cepeda, Wixted, & Rohrer, 2005), practice questions on two occasions might be expected to result in greater delayed retention than practice questions on only one occasion. Indeed, McDaniel et al. (2011) found that when feedback followed the practice questions, questions asked at the beginning and end of a lesson, compared to questions asked only at the end, resulted in better performance on a delayed review just prior to an exam.

The overall effectiveness of repeated practice questions may not be as strong as intuitively expected, however. McDaniel et al. (2011) found that on unit exams, information that was tested twice (before and after a lesson), compared to only once (after the lesson), was retained equally well. Furthermore, when information was tested on a review just prior to an exam, that review opportunity (even if it occurred just once) was often as effective as the review opportunity plus up to three prior quizzes. A strong benefit of retrieval practice occurred for material that was tested on the review—whether it appeared only on the review, or up to three times previously—relative to information that was not tested at all. Along these same lines, the current study showed that information tested at the end of class, regardless of whether it was also tested at the beginning of class, was retained better on the weekly quizzes compared to information that was not tested at all.

Thus, the current results add to a sizeable literature showing that retrieval practice enhances learning (e.g., Carpenter, 2012; Dunlosky et al., 2013; Karpicke, in press; Kornell & Vaughn, 2016; McDermott et al., 2014; Roediger, Agarwal, et al., 2011; Rowland, 2014), and to a smaller but ever-increasing literature demonstrating these effects in classrooms (Butler et al., 2014; Carpenter, Pashler, & Cepeda, 2009; Carpenter et al., 2016; Goossens, Camp, Verkoeijen, Tabbers, & Zwaan, 2014; Horn & Hernick, 2015; Jaeger, Eisenkraemer, & Stein, 2015; Karpicke, Blunt, Smith, & Karpicke, 2014; McDaniel, Anderson, Derbish, & Morrisette, 2007; McDaniel, Wildman, & Anderson, 2012; Pyburn et al., 2014; Roediger, Agarwal, et al., 2011). In the current study these benefits were observed with questions that assessed conceptual knowledge of chemical engineering, often requiring students to reason through a process by applying physical laws and principles. The effects of retrieval measured here were based on repetitions of the same questions that had been encountered previously, and so performance may have been based on students’ memories for the previously seen questions and answers. Higher-level understanding and transfer of the tested concepts was not assessed in the current study, although there is some evidence that retrieval can promote this type of knowledge as well (e.g., Butler, 2010; Carpenter, 2012).

With regards to the effects of prequestions, however, the limited research to date suggests that these effects are much smaller in the classroom than what has been observed in the laboratory. Comparisons of performance via traditional hypothesis testing revealed no significant advantage in memory for information that had been prequestioned vs. information that had not, and no significant enhancement of prequestions on the benefits of retrieval practice. This was further corroborated by the Bayesian analyses, which revealed substantial evidence in favor of the null hypothesis. Together, these results show that prequestions do not strongly and consistently affect performance in classroom settings in the way that they have been observed to affect performance in laboratory studies. The reasons for these apparently discrepant findings are currently unknown, and further research is encouraged that can shed light on the factors that can account for the variance in performance in classroom settings.

One potential explanation for the reduced effects of prequestions in classrooms could be associated with the length and complexity of information presented. Though prequestions have been shown to improve learning of fairly short reading passages (Peeck, 1970; Little & Bjork, 2016; Pressley et al., 1990; Richland et al., 2009; Rickards et al., 1976), and brief videos lasting only a few minutes (Carpenter & Toftness, 2017), they may be less likely to be effective when the information is lengthy and more complex, as this would make it harder to notice and connect the information in the lesson that is relevant to the prequestions. Questions requiring computational problem-solving, like those used in the current study, may represent a degree of complexity that makes these connections particularly challenging, such that the effectiveness of prequestions may be inherently limited for this type of material.

Indeed, if prequestions work by encouraging attention to the material (Peeck, 1970; Pressley et al., 1990), it is possible that these effects are most likely to occur under conditions where sustained attention is encouraged, such as in brief presentations of fairly simple material. Effects of prequestions, therefore, may be consistently limited in classroom environments where learning consists of higher-level concepts across a lengthy (e.g., 50 minutes or more) interval of time. In this way, instead of using prequestions to promote learning of material over an entire class period, prequestions may be better suited for brief segments of information from class, similar to the benefits of interpolated testing (Szpunar, Jing, & Schacter, 2014; Szpunar, Khan, & Schacter, 2013).

In situations where prequestions enhance learning, another reason they may do so is by providing a metacognitive reality check that reduces students’ overconfidence, in turn enhancing the effort and attention devoted to learning the material. The current study is the first known study to collect students’ confidence ratings at the time of the prequestion. After controlling for other relevant factors, we found no relationship between students’ initial confidence in their answer to the prequestion and later accuracy on that question. This result must be interpreted with caution, however, as we did not obtain an overall benefit of prequestions, and Bayesian analyses revealed only weak evidence for the lack of effects in the regression model. Further, it is possible that the metacognitive reality check may only be beneficial if the learning episode is brief enough to permit sustained effort and attention to the material, as discussed above. Future research is encouraged that explores the effects of prequestions under conditions of varying presentation durations, different instructional approaches, and individual student differences.

## Conclusions

Though prequestions have produced fairly strong effects in simplified laboratory-based environments, these effects appear to be attenuated in classroom environments. The effects of prequestions are only beginning to be explored in the classroom and will require further research to be well-understood. Notwithstanding the uncertainty of the prequestion effect in classrooms, we found that the retrieval practice effect is alive and well. Strong benefits on later learning occurred for material that was tested at the end of class and accompanied by feedback, relative to material that was not tested. Thus, when educators have limited class time, practical advice may be to withhold practice questions at the beginning of class and reserve them until after the lesson, when they are apparently most likely to be effective. These effects add to a large body of research demonstrating the power of testing to not only measure, but also potentiate, learning of course concepts.

## Additional file

Additional file 1: Data File. (XLSX 192 kb)

## Footnotes

1 To calculate the Bayes factor for the effects of question type, we used the calculator provided by Rouder, Speckman, Sun, Morey, and Iverson (2009).

## References

[CR1] Ackerman PL, Kanfer R, Beier ME (2013). Trait complexity, cognitive ability, and domain knowledge predictors of baccalaureate success, STEM persistence, and gender differences. Journal of Educational Psychology.

[CR2] Berlyne DE (1954). An experimental study of human curiosity. British Journal of Psychology.

[CR3] Berlyne DE (1962). Uncertainty and epistemic curiosity. British Journal of Psychology.

[CR4] Bjork RA, Dunlosky J, Kornell N (2013). Self-regulated learning: Beliefs, techniques, and illusions. Annual Review of Psychology.

[CR5] Bull SG, Dizney HF (1973). Epistemic curiosity arousing prequestions: Their effect on long-term retention. Journal of Educational Psychology.

[CR6] Butler AC (2010). Repeated testing produces superior transfer of learning relative to repeated studying. Journal of Experimental Psychology: Learning, Memory, & Cognition.

[CR7] Butler AC, Marsh EJ, Slavinsky JP, Baraniuk RG (2014). Integrating cognitive science and technology improves learning in a STEM classroom. Educational Psychology Review.

[CR8] Carpenter SK (2012). Testing enhances the transfer of learning. Current Directions in Psychological Science.

[CR9] Carpenter SK, Lund TJS, Coffman CR, Armstrong PI, Lamm MH, Reason RD (2016). A classroom study on the relationship between student achievement and retrieval-enhanced learning. Educational Psychology Review.

[CR10] Carpenter SK, Pashler H, Cepeda NJ (2009). Using tests to enhance 8th grade students’ retention of U. S. history facts. Applied Cognitive Psychology.

[CR11] Carpenter SK, Sachs RE, Martin B, Schmidt K, Looft R (2012). Learning new vocabulary in German: The effects of inferring word meanings, type of feedback, and time of test. Psychonomic Bulletin & Review.

[CR12] Carpenter SK, Toftness AR (2017). The effect of prequestions on learning from video presentations. Journal of Applied Research in Memory and Cognition.

[CR13] Chen X (2013). STEM attrition: College students’ paths into and out of STEM fields (NCES 2014-001).

[CR14] Dunlosky J, Rawson KA, Marsh EJ, Nathan MJ, Willingham DT (2013). Improving students’ learning with effective learning techniques: Promising directions from cognitive and educational psychology. Psychological Science in the Public Interest.

[CR15] Fazio LK, Huelser BJ, Johnson A, Marsh EJ (2010). Receiving right/wrong feedback: Consequences for learning. Memory.

[CR16] Finn B, Metcalfe J (2010). Scaffolding feedback to maximize long-term error correction. Memory & Cognition.

[CR17] Goossens NAMC, Camp G, Verkoeijen PPJL, Tabbers HK, Zwaan RA (2014). The benefit of retrieval practice over elaborative restudy in primary school vocabulary learning. Journal of Applied Research in Memory & Cognition.

[CR18] Hartwig MK, Dunlosky J (2012). Study strategies of college students: Are self-testing and scheduling related to achievement?. Psychonomic Bulletin & Review.

[CR19] Horn S, Hernick M (2015). Improving student understanding of lipids concepts in a biochemistry course using test-enhanced learning. Chemistry Education Research & Practice.

[CR20] Jaeger A, Eisenkraemer RE, Stein LM (2015). Test-enhanced learning in third-grade children. Educational Psychology.

[CR21] Jefferys H (1961). Theory of probability.

[CR22] Kang SH, Pashler H, Cepeda NJ, Rohrer D, Carpenter SK, Mozer MC (2011). Does incorrect guessing impair fact learning?. Journal of Educational Psychology.

[CR23] Karpicke, J. D. (in press). Retrieval-based learning: A decade of progress. In J. T. Wixted & J. H. Byrne (Eds.), *Learning & memory: a comprehensive reference* (Vol. 2: *Cognitive Psychology of Memory*). Academic Press.

[CR24] Karpicke JD, Blunt JR, Smith MA, Karpicke S (2014). Retrieval-based learning: The need for guided retrieval in elementary school children. Journal of Applied Research in Memory & Cognition.

[CR25] Koretsky, M. D., Falconer, J. F., Brooks, B. J., Gilbuena, D. M., Silverstein, D. L., Smith, C., …Miletic, M. (2014). The AIChE concept warehouse: A web-based tool to promote concept-based instruction. *Advances in Engineering Education, 4*, 1–27.

[CR26] Kornell N, Vaughn KE (2016). How retrieval attempts affect learning: A review and synthesis. Psychology of Learning & Motivation.

[CR27] Little JL, Bjork EL (2016). Multiple-choice pretesting potentiates learning of related information. Memory & Cognition.

[CR28] McAndrew M, Morrow CS, Atiyeh L, Pierre GC (2016). Dental student study strategies: Are self-testing and scheduling related to academic performance?. Journal of Dental Education.

[CR29] McDaniel MA, Agarwal PK, Huelser BJ, McDermott KB, Roediger HL (2011). Test-enhanced learning in a middle school science classroom: The effects of quiz frequency and placement. Journal of Educational Psychology.

[CR30] McDaniel MA, Anderson JL, Derbish MH, Morrisette N (2007). Testing the testing effect in the classroom. European Journal of Cognitive Psychology.

[CR31] McDaniel MA, Wildman KM, Anderson JL (2012). Using quizzes to enhance summative assessment performance in a web-based class: An experimental study. Journal of Applied Research in Memory & Cognition.

[CR32] McDermott KB, Arnold KM, Nelson SM, Perfect TJ, Lindsay DS (2014). The testing effect. The SAGE handbook of applied memory.

[CR33] Michaelsen LK, Knight AB, Fink LD (2004). Team-based learning: A transformative use of small groups in college teaching.

[CR34] Morehead K, Rhodes MG, DeLozier S (2016). Instructor and student knowledge of study strategies. Memory.

[CR35] Pashler H, Cepeda NJ, Wixted JT, Rohrer D (2005). When does feedback facilitate learning of words?. Journal of Experimental Psychology: Learning, Memory, & Cognition.

[CR36] Pashler, H., Bain, P. M., Bottge, B. A., Graesser, A., Koedinger, K., McDaniel, M., …Metcalfe, J. (2007). Organizing instruction and study to improve student learning. IES Practice Guide. NCER 2007–2004. National Center for Education Research. http://eric.ed.gov/?id=ED498555

[CR37] Peeck J (1970). Effect of prequestions on delayed retention of prose material. Journal of Educational Psychology.

[CR38] Pressley M, Tanenbaum R, McDaniel MA, Wood E (1990). What happens when university students try to answer prequestions that accompany textbook material?. Contemporary Educational Psychology.

[CR39] Pyburn DT, Pazicni S, Benassi VA, Tappin EM (2014). The testing effect: An intervention on behalf of low-skilled comprehenders in general chemistry. Journal of Chemical Education.

[CR40] Richland LE, Kornell N, Kao LS (2009). The pretesting effect: Do unsuccessful retrieval attempts enhance learning?. Journal of Experimental Psychology: Applied.

[CR41] Rickards JP, Anderson MC, McCormick CB (1976). Processing effects of common-word and number questions inserted in reading materials. The Journal of Educational Research.

[CR42] Roediger HL, Agarwal PK, McDaniel MA, McDermott KB (2011). Test-enhanced learning in the classroom: Long-term improvements from quizzing. Journal of Experimental Psychology: Applied.

[CR43] Roediger HL, Putnam AL, Smith MA, Mestre JP, Ross BH (2011). Ten benefits of testing and their applications to educational practice. The psychology of learning and motivation: Cognition in education.

[CR44] Roediger HL, Pyc MA (2012). Inexpensive techniques to improve education: Applying cognitive psychology to enhance educational practice. Journal of Applied Research in Memory & Cognition.

[CR45] Rouder JN, Morey RD (2012). Default Bayes factors for model selection in regression. Multivariate Behavioral Research.

[CR46] Rouder JN, Speckman PL, Sun D, Morey RD, Iverson G (2009). Bayesian t-tests for accepting and rejecting the null hypothesis. Psychonomic Bulletin & Review.

[CR47] Rowland CA (2014). The effect of testing versus restudy on retention: A meta-analytic review of the testing effect. Psychological Bulletin.

[CR48] Sibley J, Ostafichuk P, Roberson B, Franchini B, Kubitz KA (2014). Getting started with team-based learning.

[CR49] Szpunar KK, Jing HG, Schacter DL (2014). Overcoming overconfidence in learning from video-recorded lectures: Implications of interpolated testing for online education. Journal of Applied Research in Memory & Cognition.

[CR50] Szpunar KK, Khan NY, Schacter DL (2013). Interpolated memory tests reduce mind wandering and improve learning of online lectures. Proceedings of the National Academy of Sciences.

